# Prevalence and factors associated with recent intimate partner violence and relationships between disability and depression in post-partum women in one clinic in eThekwini Municipality, South Africa

**DOI:** 10.1371/journal.pone.0181236

**Published:** 2017-07-20

**Authors:** Andrew Gibbs, Bradley Carpenter, Tamaryn Crankshaw, Jill Hannass-Hancock, Jennifer Smit, Mark Tomlinson, Lisa Butler

**Affiliations:** 1 Gender and Health Research Unit, South African Medical Research Council, Pretoria, South Africa; 2 Health Economics and HIV/AIDS Research Division (HEARD), University of KwaZulu-Natal, Durban, South Africa; 3 HIV-Prevention Research Unit, South African Medical Research Council, South African Medical Research Council, Durban, South Africa; 4 MatCH Research Unit, Department of Obstetrics and Gynaecology, Faculty of Health Sciences, University of the Witwatersrand, Johannesburg, South Africa; 5 Department of Psychology, Stellenbosch University, Stellenbosch, South Africa; 6 Institute for Collaboration on Health, Intervention and Policy, University of Connecticut, Storrs, Connecticut, United States of America; University of Washington, UNITED STATES

## Abstract

Intimate partner violence (IPV) experienced by pregnant and post-partum women has negative health effects for women, as well as the foetus, and the new-born child. In this study we sought to assess the prevalence and factors associated with recent IPV amongst post-partum women in one clinic in eThekwini Municipality, South Africa, and explore the relationship between IPV, depression and functional limitations/disabilities. Past 12 month IPV-victimisation was 10.55%. Logistic regression modelled relationships between IPV, functional limitations, depressive symptoms, socio-economic measures, and sexual relationship power. In logistic regression models, overall severity of functional limitations were not associated with IPV-victimisation when treated as a continuous overall score. In this model relationship power (aOR0.22, p = 0.001) and depressive symptoms (aOR1.26, p = 0.001) were significant. When the different functional limitations were separated out in a second model, significant factors were relationship power (aOR0.20, p = 0.001), depressive symptoms (aOR1.20, p = 0.011) and mobility limitations (aOR2.96, p = 0.024). The study emphasises that not all functional limitations are associated with IPV-experience, that depression and disability while overlapping can also be considered different drivers of vulnerability, and that women’s experience of IPV is not dependent on pregnancy specific factors, but rather wider social factors that all women experience.

## Background

IPV during pregnancy and in the post-partum period is relatively common globally [1]. The prevalence of IPV during pregnancy varies widely across settings. A review of studies in Africa found IPV prevalence during pregnancy ranging from 2% to 57%, with a meta-analysis estimating an overall prevalence of 15.23%, which included physical, sexual and emotional violence [2]. In a clinic setting in Durban, South Africa, 5.2% of women in antenatal care experienced physical and/or sexual IPV in the past year [3]. However, this is a relatively low rate of reporting given the wider prevalence of IPV in South Africa [4, 5], where population-based studies show lifetime physical IPV-victimisation prevalence of 33%, with a past-year prevalence of 13% [6].

Experiencing IPV is not only a human rights violation, but also has profound health and social consequences for women [7]. Women who experience IPV are more likely to be depressed [8] and have greater physical injuries [7]. IPV-victimisation, that is experiencing IPV, is also associated with greater alcohol and drug use [9]. Additionally, in southern and eastern Africa, women who experience IPV are more likely to acquire HIV [10].

Experiencing IPV during pregnancy and in the post-partum period has not only serious health and social consequences for women, but also negative implications for the developing foetus and the new-born child, such as higher levels of pre-term birth and lower birth weight compared to women not exposed to violence during pregnancy [7].

Research investigating the factors driving women’s experiences of IPV have been linked to gender inequalities, whereby women have less social, economic and political power than men and this social and economic dependency places them at risk of IPV-victimisation [11, 12]. For individual women factors associated with lower socio-economic status that place them as vulnerable to IPV-victimisation include, less education, fewer assets and lower earnings [13, 14]. Studies also demonstrate that women who experience controlling behaviours from their partner are significantly more likely to experience IPV than those who do not [15].

Women may experience additional vulnerabilities to IPV-victimisation during pregnancy. One risk factor is whether the pregnancy was unintended [2]. In addition, as women may be diagnosed with HIV for the first time during antenatal care, disclosure of their HIV-positive status can also be a factor increasing risk of IPV during pregnancy [16, 17].

Although depression is a potential consequence of IPV it has also been identified as a risk factor for experiencing IPV [8]. Recent South African modelling of the relationship between depression and IPV highlights that poor mental health, including depression, is a pathway to IPV victimisation [18], suggesting depression needs to be considered as a risk factor, as well as consequence of IPV.

In addition, recent research also suggest a strong association between IPV and disability, a factor that has not been widely investigated in mainstream IPV research. A global systematic review on violence among people with disabilities highlighted that this population are more likely to experience IPV than people without disabilities [19, 20]. This relationship is likely bi-directional, with IPV leading to disabilities (including injuries due to severe forms of IPV), and people with disabilities, in particular people with mental health conditions and intellectual disabilities, being at higher risk of IPV. Research in Africa also indicates that women with disabilities are at particular risk of experiencing all forms of violence [21, 22]. Women with disabilities’ greater vulnerability to IPV may be linked to their reduced economic power, high levels of dependency on others (for some), significant barriers to reporting violence and lack of prosecution of perpetrators [19, 20, 23]. The review also highlighted that robust evidence on IPV and disability is still largely absent, particularly in low and middle income countries, and there is a lack of evidence about the specific forms of disability that may be associated to an increase in women’s experiences of IPV [20, 24].

Additionally there may be a complex relationship between disability and depression. Depression is a health condition that can lead to disability through limitations in activities and participation in society [25]. Work amongst people living with HIV in South Africa has highlighted already the close associations between depression and disability [25]. However, there is no literature that investigates if and how the interrelationship between disability and depression may be associated to IPV.

In this paper, we sought 1) to understand the prevalence and factors associated with IPV amongst post-partum women in one clinic in eThekwini Municipality, South Africa. And 2) to understand the associations between IPV and, depression, and disability in this population amongst women experiencing IPV and those not.

## Methods

### Study design, site and population

This was a cross-sectional study, conducted in one public sector primary healthcare facility serving a large, urban informal settlement in eThekwini Municipality, South Africa. The community is characterized by dense informal dwellings, high levels of poverty, lack of access to formal sanitation and water, and generalized high levels of violence. In addition, 41% of women attending antenatal care in eThekwini Municipality, are estimated to be living with HIV [26].

All participants were aged 18 or older and were biological mothers of infants six weeks old or younger. Women were consecutively recruited from January to March 2015. Within the clinic all women were screened (if they consented) while waiting in a queue to be seen by the health provider. If women agreed and met the eligibility criteria, they were directed to be interviewed by research assistants after their clinic visit. All enrolled participants were given ZAR100 as remuneration for their time and transport. All participants provided written informed consent.

Ethical clearance for the study was obtained from the Biomedical Research Ethics Committee at the University of KwaZulu-Natal (BE397/13), Boston Children’s Hospital Ethics Review Board (IRB-P00010899), and the University of the Witwatersrand Human Research Ethics Committee (M140426). Additional approvals were obtained from the Provincial Health Research Committee in the KwaZulu-Natal Provincial Department of Health, the eThekwini District Department of Health, and the health facility where the research was conducted.

Face-to-face structured interviews using a quantitative questionnaire were conducted by female research assistants in a private space at the clinic. All interviews were conducted in isiZulu. Data were collected and entered by research staff on handheld computer tablet operating Open Data Kit (ODK) Collect. Procedures to promote data quality included skip patterns, range limits and logical checks built into the ODK Collect entry programme, as well as data validation. Participants could choose not to answer questions.

### Measures

Structured questionnaires were used to collect data. Sociodemographic questions included age and education level (including none and post-secondary). HIV-status was based on self-report, with possible responses being: HIV-positive, HIV-negative, refuse to answer, or not known. In the analysis, we recoded refused to answer and not known together as a combined category.

Socio-economic status was assessed using a range of measures. Household food insecurity was assessed using the Household Food Insecurity Access Scale (HFIAS) [27]. The HFIAS includes nine questions about food insecurity in the past thirty days. A simple sum score was used to generate a continuous variable for food insecurity where larger scores indicated greater insecurity (α = 0.85).

For assets, participants were asked whether households had 15 specific items including a television, hot running water and an electric stove. These items were combined into a scale using Principal Components Analysis of the covariance matrix to create a single score measuring household wealth. This approach is common within economic literature [28].

The primary outcome for this analysis was women’s recent physical and/or sexual IPV victimisation using a scale based on the World Health Organization’s (WHO) violence against women survey and modified for South Africa [29, 30]. Physical IPV was assessed through asking women four questions about their experience of physical IPV in their lifetime, with a typical item being “How many times has your current or any previous boyfriend, husband, or partner threatened to use or actually used a gun, knife, or other weapon against you?” Respondents could respond “never, once, more than once, or refuse to answer”. If a respondent answered affirmatively to one or more of these they were asked a single question (binary answer) of: “Has your current or any other boyfriend, husband, or partner done any of these things in the last 12 months?” The same process was used for sexual IPV with four items and then a binary for the past 12 months. A woman was classified as having experienced recent IPV if she responded positively to either or both questions for past 12 month sexual or physical IPV. This approach has been previously used in South Africa [29].

Disability was understood as related to functional limitations and assessed using the 12 item WHODAS 2.0 (α = 0.81) [31]. The WHODAS 2.0 measures functional limitations (and potential disability) in six domains (cognition, mobility, self-care, getting along, participation, and managing life activities). Items prompt activities within the last 30 days (e.g. “In the past 30 days, how much difficulty did you have in taking care of your household responsibilities?”). Responses were on a five-point Likert-type item including categories of none, mild, moderate, severe/extreme difficulties, and “cannot do at all”. Two items were asked for each domain and the scale is reweighted as both a continuous scale, and also into the six categorical variables for each domain. The WHODAS2.0 does not include a cut-off point for classifying people as disabled. For this we used approaches from the literature including a cut of one or larger (after reweighting) to indicate the onset of functional limitations and potential disability [25, 31].

Power in a sexual relationship was measured with fourteen items of the Sexual Relationship Power Scale (SRPS) (α = 0.86) [10]. Questions had a four point Likert type response. Overall 22 (8%) participants had refused to answer at least one item of this scale. As such, we calculated the mean of the scale for individuals, rather than a direct sum, to retain a larger sample. There was no indication that people who had missing data had significantly different mean scores for the SRPS than those with no missing data (S1 Fig, S1 File).

Depressive symptoms were assessed using the PHQ-9 scale (α = 0.75), which has been previously validated in South Africa [32, 33]. Nine items ask about symptoms of depression. We treated the PHQ-9 as a continuous score [32].

### Data analysis

Analyses were conducted in STATA/IC14 with individuals as the unit of analysis. Descriptive statistics were first calculated comparing those who had not experienced recent IPV with those who had on all variables. We then undertook unadjusted logistic regression to estimate odds ratios and p-values for each secondary variable.

Due to small sample size, in the logistic regression models we entered variables that were significant in the unadjusted analyses at p<0.2. We controlled for age group and educational level. Manual backwards elimination was used to remove variables not making a statistically significant contribution to the model. The backward method is recommended because it is less likely to incur Type II error [34]. We continued this until variables were significant at p<0.05.

To understand intersections between IPV and disability, we built three separate logistic regression models. In model one, we treated WHODAS 2.0 as a continuous reweighted variable [31] to assess overall severity of functional limitations. In model two, we assessed the contribution of each form of functional limitation to account for the fact that each form may have a different direction of effect on any specific outcome [25]. To do this we separated the WHODAS 2.0 into its six domains: cognition (learning, concentrating), mobility (standing, walking), self-care (washing, getting dressed), getting along (maintaining friendships, dealing with people), life activities (work/school), and participation (joining community activities, emotional effects). Each domain was weighted as per recommendations [25] and then a dichotomous variable was created for each domain using a score of 1 or more to indicate a functional limitation [25]. This enables associations between specific functional limitations and an outcome to be identified, but cannot say anything about the overall relationship between the severity of functional limitation/disability and an outcome. In model 3, we took model two’s final regression and then assessed whether there was an interaction effect between depression and mobility limitation.

## Results

Between January 2015 and March 2015, women presenting for post-natal care at the clinic were consecutively recruited and screened for eligibility. Of 346 women approached, all agreed to be screened, of whom 310 (89.6%) met study eligibility criteria. Of those eligible, 25 (8.1%) refused to participate in the study citing lack of time. An additional 10, who initially agreed to participate, did not complete the interviews for a variety of reasons including being referred to another clinic during the study visit and thus not completing interviews, and lack of time.

In total, 275 mothers were recruited into the study, mean age 26.3 years (95%CI 25.7–27.0) (Table 1). The majority were Black (98.55%), and most (90.21%) reported having at least some secondary education (Table 1). About thirty-nine percent reported they were living with HIV (Table 1).

**Table 1:** Descriptive statistics.

		n(%)
Age	18–19	31(11.27)
	20–24	85(30.91)
	25–29	81(29.45)
	30–34	49(17.82)
	>35	29(10.55)
Race	Black	271(98.55)
	Indian	3(1.09)
	Coloured	1(0.36)
	White	0(0.00)
Education		
	Primary or Less	25(9.09)
	Secondary	242(88.00)
	Post-secondary	8(2.91)
HIV-Status	Negative	158(57.45)
	Positive	107(38.91)
	Refused to answer	10(3.64)
IPV past 12m	No	246(89.45)
	Yes	29(10.55)

Prevalence of past 12-month sexual and/or physical IPV was 10.55% (n = 29). In unadjusted analyses (Table 2), having more social support and more power in sexual relationships were associated with a reduction in women’s IPV victimisation. Women reporting higher levels of perceived family support during the pregnancy reported less IPV (OR = 0.89 [0.81–1.00], p<0.05), as did women with greater power in their sexual relationship (OR = 0.16 [0.07–0.37], p<0.0001). In contrast, the odds of IPV were significantly increased among women reporting more depressive symptoms (OR = 1.31 [1.15–1.50], p<0.0001).

**Table 2:** Descriptive relationships between recent physical and/or sexual IPV and secondary variables.

		IPV—No	IPV—Yes		
*Socio-Demographics*		Mean(95%CI)/ n(%)	Mean(95%CI)/ n(%)	OR	p-value
	Age: mean	26.3(25.6–27.0)	26.0(24.2–27.8)	0.99	p = 0.78
	Education: Primary or Less	23(92.0)	2(8.00)	base	
	Secondary	217(89.67)	25(10.33)	1.32	0.71
	Post-secondary	6(75.00)	2(25.00)	3.83	0.22
	HIV-Positive (yes)	97(90.65)	10(9.35)	0.86	0.71
	HIV status: Refused to answer	8(80.00)	2(20.00)	2.07	0.38
	Preganancy Unplanned	206(83.74)	28(96.55)	0.18	0.101
*Livelihoods- food secuirty*	* *				
Hunger >more hunger	Mean	4.74(4.20–5.28)	5.38(3.58–7.18)	1.03	0.45
Assets > more assets	Mean	1.48(1.38–1.58)	1.21(0.95–1.47)	0.63	0.076
Any social grant	Yes	202(90.58)	21(9.42)	0.57	0.21
					
*Social Support*, *Mental Health & Gender*	* *				
PHQ9 (sum)	Mean	3.35(3.02–3.68)	6.09(4.61–7.52)	1.31	p<0.0001
Perceived family support >more	Mean	16.06(15.64–16.48)	14.69(13.27–16.11)	0.89	p<0.05
Perceived community support >more	Mean	10.13(9.73–10.54)	10.41(9.16–11.67)	1.02	0.66
Sexual relationship power > is more	Mean	3.05(2.98–3.11)	2.57(2.38–2.77)	0.16	p<0.0001
					
*WHODAS—disability*					
WHODAS full scale (adjusted)	Mean	2.81(2.32–3.29)	5.79(3.65–7.94)	3.47	p<0.01
WHODAS Communication	Yes	47(19.11)	9(31.03)	1.91	0.136
WHODAS Mobility	Yes	94(38.21)	20(68.97)	3.59	<0.01
WHODAS Self-Care	Yes	10(4.07)	4(13.79)	3.78	<0.05
WHODAS getting along with people	Yes	34(13.82)	10(34.48)	3.28	<0.01
WHODAS life activities	Yes	59(23.98)	13(44.83)	2.58	<0.05
WHODAS participation	Yes	96(39.02)	18(62.07)	2.56	<0.05

Functional limitations/disability were also associated with a greater likelihood of IPV victimisation. With the reweighted WHODAS 2.0 scale, women reporting higher severity of functional limitations reported more IPV victimisation (OR3.47 [1.05–1.22], p<0.01). Women reporting challenges around self-care (OR = 3.79, p<0.05), mobility challenges (OR = 3.59, p<0.01), getting along with people (OR = 3.28, p<0.01), challenges around general life activities (OR = 2.58, p<0.05) and participation in social activities (OR = 2.56, p<0.05) all reported increased odds of IPV-victimisation.

**Fig 1:**
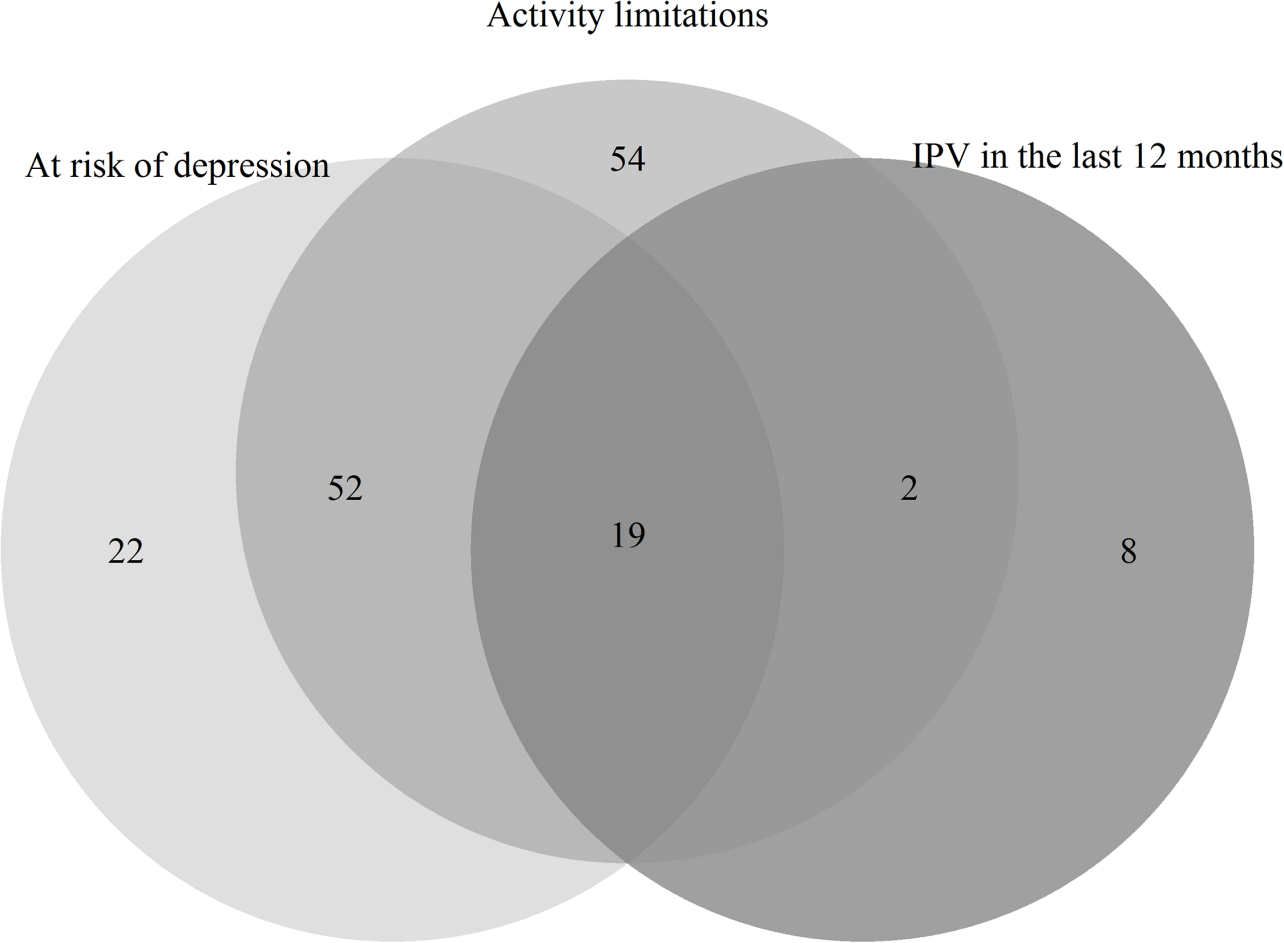
Venn diagram showing overlaps between physical and/or sexual IPV, depressive symptoms, and functional limitations.

To understand the potentially complex relationship between IPV, depressive symptoms, and disability we examined data using two methods. Firstly, our descriptive analysis in the Venn Diagram (Fig 1) highlights the overlap between IPV, depressive symptoms, and any functional limitation/disability. A large portion of those reporting functional limitations/disability (n = 127), 55.91%, also reported mild or greater depressive symptoms (n = 71). There was also a strong overlap between IPV, depressive symptoms, and functional limitations in the Venn Diagram, with 72% (n = 21) of women who reported experiencing IPV also reporting either depressive symptoms and/or a functional limitation/disability. Almost all women who reported experiencing IPV *and* depressive symptoms also reported functional limitations, only 1% (n = 2) did not report this. This suggests a high degree of overlap between depressive symptoms and potential disability in this population.

In model 1 (Table 3), where functional limitations were treated as a continuous reweighted variable, the adjusted model showed only two factors significantly associated with IPV-victimisation. Women reporting more power in their sexual relationship had less IPV-victimisation (aOR = 0.22, p = 0.001), while higher depressive symptoms were associated with more IPV (aOR = 1.26, p = 0.001). The pseudo R2 for the model was 0.19.

**Table 3:** Factors associated with recent physical and/or sexual IPV in regression.

			Pseudo R2 = 0.19
**Model 1**	aOR	p-value	95% CI
SRP (> = more power in relationship)	0.22	0.001	0.09–0.56
PHQ score (> = more depressed)	1.26	0.001	1.10–1.44
			
**Model 2**			Pseudo R2 = 0.22
SRP (> = more power in relationship)	0.20	0.001	0.07–2.07
PHQ score (> = more depressed)	1.20	<0.01	1.04–1.38
Mobility limitations	2.96	<0.05	1.15–7.57
**Model 3**	** **	** **	Pseudo R2 = 0.22
SRP (> = more power in relationship)	0.19	0.001	0.07–0.51
PHQ score (> = more depressed)	1.11	0.49	0.82–1.51
Mobility limitations	1.93	0.47	0.32–11.58
Interaction PHQ mobility limitations	1.1	0.59	0.78–1.54

All models in Table 3 control for age and education

In model 2 (Table 3) functional limitations were treated as separate categorical variables. As with model 1, women with more power in their sexual relationship experienced less IPV (aOR = 0.20, p = 0.001). Higher depressive symptoms were associated with greater odds of experiencing IPV (aOR = 1.20, p = 0.001). In addition, women reporting having mobility limitations reported more IPV-victimisation (aOR = 2.96, p = 0.024). For the model pseudo R2 = 0.22.

In model 3 (Table 3) an interaction effect was estimated in the final model for the relationship between depression and mobility limitations. Despite all adjusted odds ratios showing a positive relationship, the interaction was not significant, nor were its principle components.

## Discussion

Recent IPV in postpartum women in this sample was relatively high at 10.55%. This is higher than a study by Groves, McNaughton-Reyes [3] in South Africa which reported that 5.2% experienced physical and/or sexual IPV during pregnancy and in the post-partum period. However, this is lower than the estimated prevalence of IPV experienced by pregnant women in Africa of 15.23%, although this estimate also included emotional violence which is more commonly reported [2]. However, the rates of violence, while similar to population-based estimates in South Africa [4], is also relatively low, given that the clinic served a large informal settlement, and studies suggest women living in informal settlements are at high risk of experiencing IPV [29].

There appears to be a complex relationship between IPV and functional limitations/disability. From other research it is known that people with disabilities are substantially more likely to experience violence than people without disabilities [20]. The mean WHODAS2.0 score was much higher among women experiencing IPV then those not experiencing IPV. However in model 1, increasing overall severity of functional limitations had no impact on the likelihood of IPV-victimisation. In model two, having a functional mobility limitation did increase the likelihood of IPV-victimisation (aOR = 2.96, p = 0.024).

These results suggest that not all forms of functional limitations have the same risk of increasing women’s experiences of IPV in this sample. In the second model, which focused on categorising women into different forms of functional limitations, only those with mobility limitations experienced higher IPV vulnerability in the final model. As such, it is only women with the specific mobility limitations who experienced greater likelihood of IPV-victimisation. This relationship is likely to be bi-directional, with women who experience IPV having reduced mobility due to physical harm, and women with reduced mobility being more vulnerable to IPV victimisation. This has been seen in other studies, but the direction of causality is unclear [25]. In this study there was an almost complete overlap between depression and functional limitations (Venn diagram and data not shown). This could be due to the bi-directionality or some other underlying relationship between depression and disability.

In model three there was no significant interaction effect between depression and mobility limitations, which may be explained with the small sample size. Despite the lack of interaction effect and, even though we don't yet understand the direction of these associations, our study provides a clear indication that depression and functional limitations should not be considered in isolation, but that the combination of both may create a further risk for IPV. Depression can be associated with certain life experiences that are more common among people with disabilities, who face many unique problems and challenges, which may place them at increased risk for depression (mobility limitations accessibility issues, social barriers and isolation, dependency, unemployment and poverty, lack of relationship power).

As with other studies, women’s power in sexual relationships was an important factor shaping IPV-vulnerability, whereby those with more power in relationships, experienced less violence in both models. Studies have highlighted the central role of women’s power in relationships as being protective of experiencing IPV [10, 11, 15]. This study further reinforces these findings and emphasises the importance of working to strengthen women’s power in relationships.

Finally, in both models women reporting higher levels of depressive symptoms had greater likelihood of IPV-victimisation. The relationship between depression and IPV is likely bi-directional with depression as an outcome, and a ‘cause’, of IPV [8]. For female caregivers, depression in the post-natal period has negative impacts on children, with children displaying poorer cognitive and emotional development from an early age [35, 36]. As such, there is an important role for screening and treating for depression amongst this population.

The study showed no pregnancy ‘specific’ factors linked to IPV-victimisation. Other research has highlighted that women who have unintended pregnancies are more likely to experience IPV [2], although women who experience IPV are also more likely to have unintended pregnancies [37], so there is a lack of clarity about directionality. There was some indication that there were pregnancy specific factors shaping IPV experience, specifically, women who perceived greater family support for the pregnancy had a reduced chance of experiencing IPV in descriptive analysis. But this did not remain significant in any of the models. This suggests that women’s experience of IPV is not dependent on pregnancy specific factors, but rather wider social factors that all women experience.

This study has several limitations. First, the relatively small sample size and exploratory nature of the analysis meant that associations might have been too small to identify. In addition, as it was a clinic-based sample, it likely under-sampled those with the most severe forms of disability and depression, and cannot be thought of as generalizable to the wider population, although the relationships may still hold true. Finally, as the study is cross-sectional, the directionality of the relationships between IPV, the onset of disability, and depression cannot be established.

## Conclusions

Few studies have explored the relationship between IPV, depression and disability. This analysis highlights the complex relationship between these issues and raises questions about how to conceptualise and analyse these relationships. First, it showed that while there was a close overlap between depression and disability in the study, they also appeared to be distinct in their relationship to IPV-victimisation. Second, it highlighted that not all forms of disability appeared to place women at risk of IPV-victimisation, but rather it was specific forms of disability, in this case mobility limitations that were associated to women’s vulnerability. As such, research needs to follow women over time to understand the interaction of depression and disability. Analysis also needs to disaggregate effects via disability types to understand when and how different functional limitations may impact on IPV-victimisation.

The findings emphasise several important approaches for developing interventions to prevent IPV-victimisation. It is important to recognise that pregnant women are vulnerable to IPV because of wider social factors, rather than pregnancy specific factors alone. Thus, strengthening women’s power in relationships, and working to reduce depressive symptoms, should be an important component of interventions. There is a substantive and growing body of research emphasising the importance of women’s social empowerment [12] and these interventions need to be adapted and implemented for pregnant women during the ante-natal and post-natal period. Similarly, screening for depression in the post-partum period may also have an impact on IPV victimisation.

The study also highlights a strong relationship between certain forms of disability and the risk of IPV-victimisation. Given the lack of effective interventions to reduce IPV amongst women living with disabilities, adapting current evidence-based approaches so that they also include women with more severe disabilities remains a critical challenge to ensure that all women can live free from violence [24].

## Supporting information

S1 Fig Box plot showing SRPS means for missing data. Mean for SRPS data for participants with no missing data, and for participants with missing data.
(TIF)

S1 File Treatment of missing SRPS data. (DOCX)

S2 File Underlying data for analysis. Individual level participant data used in analysis.
(XLS)
