# Supplementary figures and images for: Prevalence and factors associated with recent intimate partner violence and relationships between disability and depression in post-partum women in one clinic in eThekwini Municipality, South Africa
Source: PLoS One. 2017 Jul 20;12(7):e0181236. doi: 10.1371/journal.pone.0181236 (PMC5519063; doi:10.1371/journal.pone.0181236)

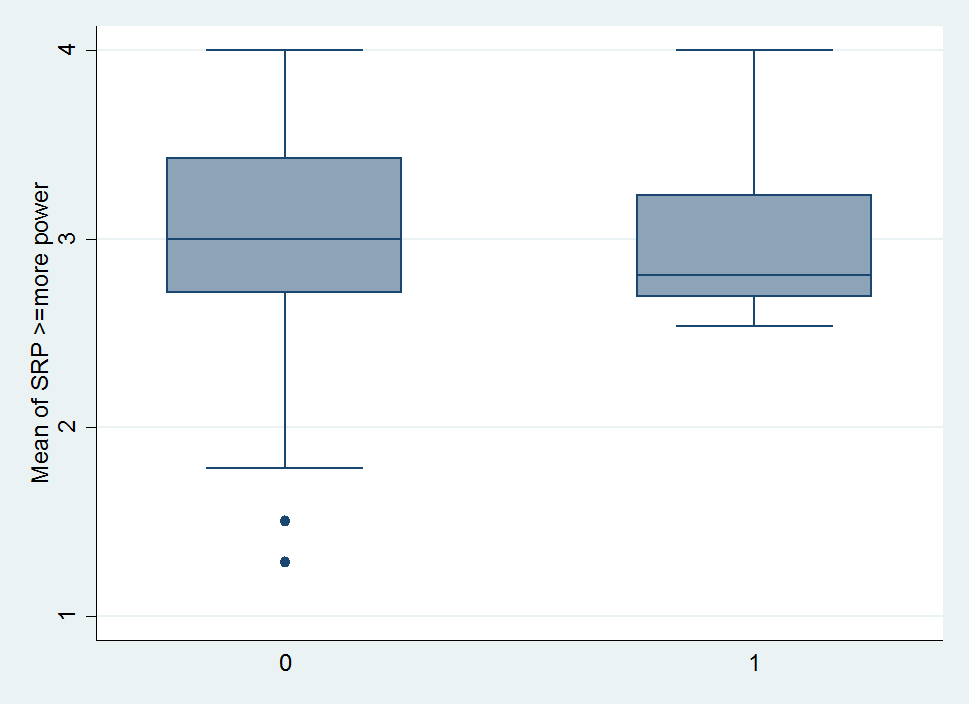

Supplement: S1 Fig — Mean for SRPS data for participants with no missing data, and for participants with missing data. (TIF) [file pone.0181236.s001.tif]
