# Supplementary material for: Prevalence and factors associated with recent intimate partner violence and relationships between disability and depression in post-partum women in one clinic in eThekwini Municipality, South Africa
Source: PLoS One. 2017 Jul 20;12(7):e0181236. doi: 10.1371/journal.pone.0181236 (PMC5519063; doi:10.1371/journal.pone.0181236)
Supplement: S1 File — (DOCX) [file pone.0181236.s002.docx]

**Supplementary file**

The SRPS comprised of fourteen items in this study. Seventeen participants had one item missing, four had two items missing, and one had three items missing. To deal with missing data on the sexual relationship power scale we took a mean score of the items that had been completed per participant.

There was no significant difference between those with missing data and those without missing data in terms of overall SRPS mean score. We created a boxplot for visual inspection (S1 Fig). In addition a t-test showed no significant differences (p= 0.64).

S1 Fig: Box plot comparing mean scores of SRPS between those with missing data and those without missing data
